# Prevalence and Correlates of Disposable E-Cigarette Use Among Adolescents and Young Adults in Southern Poland

**DOI:** 10.3390/jcm15135318

**Published:** 2026-07-07

**Authors:** Józefa Dąbek, Julia Żerdka, Patryk Brasse, Grzegorz Smolka

**Affiliations:** 1 Department of Cardiology, Faculty of Health Sciences in Katowice, Medical University of Silesia, 40-055 Katowice, Poland; gmolka@sum.edu.pl; 2 Student Scientific Club, Department of Cardiology, Faculty of Health Sciences in Katowice, Medical University of Silesia, 40-055 Katowice, Poland; julia.zerdka@gmail.com (J.Ż.); brassepatryk@gmail.com (P.B.)

**Keywords:** disposable e-cigarettes, addiction, nicotine dependence

## Abstract

**Introduction:** In recent years, electronic cigarettes, including disposable e-cigarettes and tobacco heating devices, have grown in popularity, especially among young people and young adults. Although e-cigarettes are touted as a “safer” alternative to traditional cigarettes, a growing body of evidence indicates that vaping can cause serious health problems, especially in the respiratory and cardiovascular systems. **Aim of the Study:** The aim of the study was to assess the prevalence of disposable e-cigarettes and other nicotine products among adolescents and young adults, as well as to examine the factors associated with regular smoking and nicotine dependence across individual nicotine-containing products. **Material and Methods:** The survey was conducted among residents of southern Poland. A total of 625 (100%) people were examined. Among them were: 350 women and 275 men. The subjects were aged between 14 and 29 years (Median and 25th–75th percentile: 19; 16–23). The study used an original questionnaire, the completion of which was anonymous and voluntary. The questions included in the survey concerned the smoking status of the respondents, the main sources of nicotine intake and general information such as age, gender, place of residence, employment or education, and financial stability of the family. The survey used the Fagerström test to examine the degree of nicotine dependence among the subjects. **Results:** A total of 34% (212; 33.92%) of the surveyed group of adolescents and young adults were smokers, most of whom used disposable e-cigarettes (69; 32.55%). In addition, 60% (368; 58.88%) of respondents reported having tried disposable e-cigarettes. Smokers, of both traditional cigarettes and e-cigarettes, accounted for 14% (88; 14.08%) of the study group. The results of the study showed a statistically significant effect of the age of the participants on the method of nicotine intake (*p* = 0.013). In the younger group, e-cigarettes were more often used, while in the older group, the use of heated tobacco products and traditional cigarettes dominated. Respondents’ median age at the first use of nicotine products was 16 years. Factors such as living in the city, lower financial stability, and having a parent who smoked during childhood were significantly associated with a higher likelihood of regular nicotine product use. Among the regular users, a high level of nicotine dependence was observed in 28.64% (*n* = 61) of individuals, with the largest proportion occurring among those using electronic cigarettes (39.37%; *n* = 50). **Conclusions:** 1. In the study group of adolescents and young adults, disposable e-cigarettes were the most used nicotine-containing product. 2. Parental smoking and male gender were positively associated with the likelihood of smoking, whereas primary education and financial stability were inversely associated with tobacco use. 3. Electronic cigarette users exhibited the highest average nicotine dependence scores. Multivariate analysis confirmed that male gender, the type of nicotine product used, and place of residence were independently associated with higher Fagerström test scores.

## 1. Introduction

In recent years, the model of smoking in society has changed significantly. In many countries, there has been a marked decrease in the number of people smoking traditional cigarettes. This is mainly due to greater knowledge about the harmfulness of smoking, reinforced by health campaigns and restrictive regulations on the sale and advertising of tobacco products. Electronic cigarettes and devices that heat tobacco, on the other hand, have gained popularity, especially among young people and young adults. Products have become an essential part of modern smoking culture, young people often see e-cigarettes as a less harmful alternative to traditional smoking, exposing themselves to numerous health effects [1,2].

Disposable e-cigarettes are a relatively new product on the market, which means that there is a lack of long-term research on their effects on health and their link to chronic diseases.

However, studies indicate that chemicals in e-cigarette liquids (m.in. propylene glycol, glycerol, flavorings and sweeteners) can lead to an increased risk of developing cardiovascular and respiratory diseases, which can lead to serious health consequences in the long term. When these substances are heated, harmful compounds such as formaldehyde and acrolein can be formed, which can irritate and damage lung tissue. In 2019, there were numerous cases of severe lung damage in people who use e-cigarettes, the so-called EVALI syndrome (E-cigarette or Vaping Product Use-Associated Lung Injury), i.e., lung damage associated with the use of e-cigarettes and other vaping products. EVALI syndrome was most common in people who used e-cigarettes containing THC (tetrahydrocannabinol)—a psychoactive substance found in cannabis. However, cases of EVALI have also been reported in people who use only nicotine liquids for e-cigarettes [3,4]. In turn, in people with asthma or other chronic lung diseases, vaping can worsen symptoms and lead to exacerbations of the disease [5,6]. Preliminary studies indicate that people who use e-cigarettes are more likely to experience airway damage and deteriorating lung function, which increases the risk of developing chronic obstructive pulmonary disease (COPD) [7]. In addition to the effects on the respiratory system, there is a growing body of evidence suggesting that e-cigarettes can also have a negative impact on the cardiovascular system. Vaping can increase blood pressure, increase heart rate, and cause inflammation of blood vessels, leading to the development of heart diseases such as heart attack and failure, as well as increasing the risk of stroke [8,9,10].

Although e-cigarettes are touted as a “safer” alternative to traditional cigarettes, a growing body of evidence indicates that vaping can cause serious health problems, especially in the respiratory and cardiovascular systems. Available evidence suggests that vaping is increasingly prevalent among younger populations, raising serious concerns about the long-term health consequences. Young people, whose bodies and respiratory systems are still developing, are more susceptible to the harmful effects of inhaling chemicals contained in e-cigarettes [11].

The widespread use of e-cigarettes among young people and the not fully understood risks associated with them highlight the urgent need to take action to prevent and reduce their use, especially in the age group studied. It is crucial to introduce stricter legal regulations regarding the sale and promotion of e-cigarettes, especially in the context of young people’s access to these products. Many users are not aware of the potential risks, and marketing is often directed to younger consumers, emphasizing attractive flavors and modern design, further increasing interest in these products. It is also important to educate the public, including information campaigns aimed at young people and parents, which highlight the real health risks associated with vaping. Only by increasing awareness and limiting the availability of e-cigarettes among young people will it be possible to minimize their negative impact on the health of future generations [12].

Despite the rapidly growing volume of literature on electronic cigarette use, several critical research gaps remain. First, a vast majority of existing studies rely heavily on online, web-based surveys, which are inherently prone to self-selection bias, bot completion, and a lack of environmental control during data collection. Second, there is a scarcity of data tracking the shifting patterns of nicotine consumption across the critical transitional period from mid-adolescence to young adulthood (ages 14–29) under a single, unified research protocol. Furthermore, regional variations in Central and Eastern Europe remain understudied, particularly within highly urbanized, industrialized, and densely populated areas such as Southern Poland (the Silesian region). This region serves as a crucial socio-economic and academic hub, making its youth demographic highly representative of modern urban public health trends. This study distinguishes itself from prior literature by deploying a strictly controlled, entirely in-person, paper-based questionnaire administered in classroom settings across both secondary schools and universities. By incorporating specific methodological safeguards—such as response option randomization to eliminate primacy bias—this work provides a highly reliable, regionally grounded blueprint of contemporary nicotine dependence.

## 2. Aim of the Study

The aim of the study was to assess the prevalence of disposable e-cigarettes and other nicotine products among adolescents and young adults, as well as to examine the factors associated with regular smoking and nicotine dependence across individual nicotine-containing products.

## 3. Material and Methods

### 3.1. Study Design

This cross-sectional survey was conducted between December 2024 and December 2025 among young residents of southern Poland. The study aimed to assess patterns of nicotine product use, age of nicotine initiation, and nicotine dependence among adolescents and young adults.

### 3.2. Study Setting

The study was conducted in the Silesian and Lesser Poland Voivodeships, Poland. Data were collected in person using paper-based questionnaires distributed in secondary schools and universities.

Institutions were selected using a convenience sampling approach based on geographic accessibility and willingness to participate. Of the 10 institutions initially invited, 5 secondary schools and 4 universities agreed to participate, corresponding to an institutional response rate of 90.0%.

The recruitment protocol was standardized across all participating institutions. Before questionnaire administration, researchers provided a brief explanation of terminology used in the questionnaire (e.g., differences between electronic cigarettes, disposable e-cigarettes, heated tobacco products, nicotine pouches, liquids, and vaping) to ensure accurate understanding. Participants completed anonymous questionnaires immediately after distribution during scheduled class sessions. Any questions raised by participants were clarified by the researchers without suggesting responses.

To minimize selection bias, entire classroom groups were surveyed during designated teaching hours. To avoid response priming and social desirability bias, educational lectures concerning the health effects of electronic cigarette use were provided only after all questionnaires had been completed and collected. No financial or material incentives were offered.

### 3.3. Participants

#### 3.3.1. Eligibility Criteria

Eligible participants were individuals aged 14–29 years residing in southern Poland who agreed to participate voluntarily. For participants younger than 18 years, written consent from legal guardians was obtained in addition to the participant’s assent.

Participants with substantially incomplete questionnaires or questionnaires showing careless or inconsistent responses were excluded from analysis.

#### 3.3.2. Sampling Method

Participants were recruited using a non-probability convenience sampling strategy within participating educational institutions. Recruitment targeted intact classroom groups rather than individual volunteers to minimize within-school selection bias.

According to the Statistical Office, the population of secondary school students (14–19) and young adults (up to 29 years of age) in the Silesian Voivodeship numbers approxi-mately 625,000. A representative sample for this group is 384. Our study group had 625 respondents. However, participants were recruited using a non-probability convenience sampling method. While this approach facilitated efficient data collection, it does not ensure clearly representativeness of the sample and may be associated with selection bias.

#### 3.3.3. Sample Size

A total of 650 questionnaires were distributed. Of these, 625 questionnaires were completed correctly and included in the final analyses, yielding a response rate of 96.15%. Twenty-five questionnaires (3.85%) were excluded because of substantial missing data or evidence of careless completion. The final study population consisted of 625 participants aged 14–29 years (median 19 years, interquartile range 16–23 years), including 350 women and 275 men.

### 3.4. Variables and Measurements

**Questionnaire**

Data were collected using an original structured questionnaire consisting of 99 items assessing:

- Demographic characteristics;
- Educational and employment status;
- Family financial situation;
- Smoking status;
- History of nicotine initiation;
- Current nicotine consumption patterns;
- Exposure to various nicotine products;
- Use of disposable electronic cigarettes.

The questionnaire contained both single-choice and multiple-choice questions and allowed participants to provide additional responses where appropriate. To reduce primacy bias, the order of response options was randomized across questionnaires. 

Prior to implementation, the questionnaire underwent internal review by senior academic staff and qualitative pre-testing in a convenience sample of five participants to assess face validity and clarity. The complete questionnaire is available from the corresponding author upon reasonable request.

### 3.5. Operational Definitions

The following definitions were applied throughout the study:

**Ever use**—having used any nicotine-containing product (combustible cigarettes, electronic cigarettes, heated tobacco products or nicotine pouches) at least once during lifetime.

**Regular use**—current use of nicotine-containing products on at least five days per week for a minimum of three consecutive months.

**Occasional use**—current non-daily nicotine use occurring within the previous 30 days but less than five days per week.

Prior to questionnaire administration, respondents received standardized explanations of all nicotine-related terminology used in the survey, including standard electronic cigarettes, disposable electronic cigarettes, e-liquids, heated tobacco products, and vaping, to ensure consistent interpretation of the questionnaire items.

### 3.6. Assessment of Nicotine Dependence

Nicotine dependence among regular users was evaluated using modified versions of the Fagerström Test for Nicotine Dependence (FTND), which were specifically adapted by the authors to reflect the distinct operational characteristics of electronic cigarettes and tobacco heating devices. The adapted version of the Fagerström test used in this study is based on the methodology established by Rahman AU et al. [13]. In the original validation study, this adapted scale demonstrated an acceptable level of internal consistency (Cronbach’s alpha = 0.725) and satisfactory test–retest reliability (Spearman’s rank correlation = 0.730), supporting the validity of using these modified items for assessing nicotine dependence in users of non-combustible products.

For electronic cigarette users (both disposable and standard), the traditional items were modified to focus on ‘vaping sessions’ rather than combustible cigarettes. The adapted scale consisted of six core items scored identically to the original FTND (0 to 10 points total):

1. Time to the first vaping session after waking up (within 5 min, 6–30 min, 31–60 min, or after 60 min);
2. Difficulty refraining from vaping in places where it is forbidden (yes/no);
3. Number of vaping sessions per day (10 or less, 11–20, 21–30, or 31 and more);
4. The vaping session most difficult to give up (the first one in the morning vs. any other);
5. Vaping more frequently during the first hours after waking than during the rest of the day (yes/no);
6. Vaping even when severely ill and bedridden (yes/no).

Similarly, for tobacco heating product users, the items were adapted to evaluate the frequency of device heating sessions per day using parallel phrasing (e.g., ‘number of times using the device per day’).

The cumulative scores from these six items were summed to categorize the level of nicotine dependence. While the original FTND was developed for combustible tobacco, our adapted phrasing and structural framework directly follow established approaches in literature for assessing e-cigarette dependence, such as the e-cigarette Fagerström Test for Nicotine Dependence (e-FTND). By substituting ‘cigarettes’ with ‘vaping sessions’ and aligning the frequency metrics, the conceptual integrity of the scale was maintained. 

The critical modification and methodological optimization in our tool compared to the original e-FTND lies in the measurement of daily consumption patterns. While the original e-FTND quantifies consumption based on the number of liquid refills, cartridge changes, or exact milliliters used per day, such metrics are highly volatile and impractical for users of modern disposable e-cigarettes and heated tobacco products. To address this, our instrument quantified consumption via the “number of distinct vaping sessions per day” (10 or less, 11–20, 21–30, or 31 and more). Measuring discrete usage episodes rather than fluid volume provides a far more accurate behavioral proxy for nicotine exposure, accounting for the pre-filled, continuous-use nature of disposable devices and the standardized single-session units of heated tobacco sticks.

Nevertheless, because this specific translation was applied to a mixed cohort of disposable and standard e-cigarette users, we continue to acknowledge the lack of a separate, formal validation for this exact local version as a study limitation.

The results of the subjects were summed up and classified into the appropriate group representing the degree of nicotine addiction. Next, the association of parameters such as gender, place of residence, and stage of education on the results obtained in the test was compared. To calculate statistical significance, the Mann–Whitney U test and the Kruskal–Wallis test were used.

### 3.7. Bias

Several measures were implemented to minimize potential sources of bias.

Researchers standardized participant instructions before questionnaire administration to ensure consistent understanding of terminology.

Entire classroom groups were surveyed during scheduled educational activities to reduce within-school selection bias.

Questionnaire responses were anonymous, participation was voluntary, and no incentives were offered, reducing social desirability bias.

Educational information concerning the health consequences of electronic cigarette use was provided only after questionnaire completion to avoid influencing participants’ responses.

### 3.8. Ethical Considerations

The study was approved by the Bioethics Committee of the Medical University of Silesia in Katowice (Resolution No. BNW/NWN/0052/KB/252/24, issued on 20 November 2024). All participants provided informed consent before participation. For minors, written parental or legal guardian consent was additionally obtained. Completed questionnaires were stored securely in locked cabinets and accessed only during data entry. No identifying information was collected, ensuring complete participant anonymity. All procedures were performed in accordance with relevant ethical guidelines and regulations.

### 3.9. Statistical Analysis

All methods used in the study were carried out in accordance with relevant guidelines and regulations. The data was collected and sorted in Microsoft Excel and then processed in Statistica 13.3.

The analyzed results are presented in numerical and percentage values. For the age of first use of nicotine-containing devices, the median, 25th–75th percentile and minimum value were calculated, considering gender, smoking status and method of first contact with nicotine. The studied group was divided according to age (3 groups: 14–19, 20–24 and 25–29 years) and financial stability (3 groups: less stable, stable, more stable). Next, the relationships between these factors and the dominant method of nicotine intake were analyzed. In addition, the association of gender, place of residence and education on the regularity of nicotine consumption by the respondents was examined. To investigate statistical significance, the Chi2 and U tests of Mann–Whitney and Kruskal–Wallis were used (for data not showing features of normal distribution). The null hypothesis denied the existence of a relationship between variables. The values of *p* < 0.05 were considered statistically significant.

The normality of the distribution of quantitative data was checked by the Shapiro–Wilk test. Since the tested parameters did not show the feature of normality, non-parametric tests were used in the statistical analysis.

Post hoc pairwise comparisons were performed using the Dunn’s test with Bonferroni correction for multiple testing, specifically to validate the results presented in Tables 6 and 7.

To identify independent predictors of cigarette smoking, a multivariable logistic regression analysis was performed. The results of the model are presented as adjusted odds ratios (aOR) with 95% confidence intervals (95% CI). To account for potential confounding effects and identify independent predictors of nicotine dependence, multivariate analysis was performed. We employed multiple linear regression to assess the determinants of nicotine dependence, as measured by the Fagerström test scores. The results were expressed as beta coefficients (β) with 95% confidence intervals (CI). The model included age, gender, level of education, place of residence, financial stability, and parental smoking history as independent variables to isolate their unique associations with the severity of nicotine dependence.

## 4. Results

### 4.1. General Characteristics of the Study Group

The general characteristics of the study group, considering gender, age, place of residence, financial stability, nicotine contact, current stage of education and the main source of nicotine regularly taken by the respondents, are presented in Table 1.

The majority of the respondent group were women (350; 56%) and people aged 14–19 (329; 52.64%), and the place of residence was mainly cities (379; 60.64%). The vast majority of the surveyed families were in a stable (365; 58.40%) or more stable (226; 36.16%) financial situation. Approximately 70% (440; 70.40%) of respondents have come into contact with nicotine-containing products. The majority of respondents were people with primary education (262; 41.92%) or secondary education (279; 44.64%).

A total of 34% (212; 33.92%) of the surveyed group of young people and young adults were regular smokers, most of whom used disposable e-cigarettes (69; 11.04%) and the least traditional cigarettes (35; 5.60%).

### 4.2. Occasional Contact of Subjects with Nicotine-Containing Products

The characteristics of the study group, taking into account the occasional use of nicotine-containing products, are presented in Table 2.

The subjects willingly used various nicotine products on an occasional basis. The most used product was a disposable e-cigarette (156; 24.96%). Smokers, both of traditional cigarettes and e-cigarettes, accounted for 14% (88; 14.08%) of the surveyed group, while less than 1% (5; 0.8%) of respondents used most of the available nicotine products, such as: standard and disposable e-cigarettes, heated tobacco products, cigarillos and traditional cigarettes. Overall, 60% (368; 58.88%) of respondents reported having tried disposable e-cigarettes.

### 4.3. Characteristics of the Study Group and the Association of Individual Variables with the Regular Use of Nicotine-Containing Products

Table 3 shows the association of variables such as parental smoking during child-hood, financial stability, place of residence, gender, and education with the regular use of nicotine-containing products among the respondents. To identify independent predictors of cigarette smoking, a multivariable logistic regression analysis was performed. The results of this analysis are presented in Table 4.

Factors such as: parental smoking during childhood (*p* = 0.00016), less stable financial situation (*p* = 0.01178), living in the city (*p* = 0.02008), male gender (*p* = 0.00022) and, interestingly, education (*p* = 0.00461), including both tertiary and vocational education, were significantly associated with a higher prevalence of regular nicotine product use among the respondents.

Parental cigarette smoking during childhood remained a significant independent predictor of regular smoking among respondents (aOR = 1.98; *p* = 0.0002). Furthermore, male gender was associated with a higher likelihood of nicotine product use compared to female gender (aOR = 2.13; *p* < 0.0001). In contrast, respondents reporting financial stability (aOR = 0.40; *p* = 0.01) and those with primary education levels (aOR = 0.44; *p* = 0.0004) demonstrated significantly lower odds of regular nicotine use.

## 5. Factors Associated with Nicotine Initiation Age

Table 5 presents the comparison of the age of nicotine initiation across various sub-groups, including gender, main source of nicotine intake, nicotine first contact device, and the smoking status of the parents the median, 25th–75th percentiles and minimum values, were calculated to evaluate potential associations.

The median age of first nicotine use among respondents was 16 years. Men initiated nicotine use at an earlier age than women (*p* = 0.0035). There were no statistically significant differences in the age of smoking initiation between people whose parents smoked during their childhood and those whose parents did not smoke.

## 6. Association Between the Type of Nicotine Product Used and the Severity of Dependence

Table 6 presents the distribution of the degree of nicotine dependence across individual nicotine-containing products among the subjects, while Table 7 presents the association of variables such as gender, place of residence, current stage of education, and the nicotine product used with the results obtained in the Fagerström test.

To identify the independent predictors of nicotine dependence, a multivariate linear regression analysis was performed, including gender, type of nicotine product used, education level, place of residence, age, and financial stability. The results, adjusted for all included variables, are presented in Table 8.

In total, 61 (28.64%) respondents who regularly used nicotine-containing products demonstrated a high level of nicotine dependence to this substance, of which the largest group were e-cigarette users (50; 39.37%). The lowest number of respondents demonstrating high nicotine dependence was in the group of people who smoked cigarettes (4; 11.11%). Post hoc pairwise comparisons using Dunn’s test with Bonferroni correction showed significant differences between electronic cigarette users and conventional cigarette smokers (*p* = 0.0015) and between electronic cigarette users and heated tobacco users (*p* = 0.0049), whereas no significant difference was found between heated tobacco users and conventional cigarette smokers (*p* = 0.248).

Men in the Fagerstrom test scored an average of 5.1, while women scored 3.41. Among the users of individual nicotine-containing products, the highest average scores were obtained by respondents using e-cigarettes. The current stage of education was also significantly associated with the cumulative dependence scores.

Post hoc pairwise comparisons using Dunn’s test with Bonferroni correction were performed. The analysis demonstrated that Fagerström nicotine dependence scores were significantly higher among e-cigarette users compared to both combustible cigarette users (*p* = 0.0004) and heated tobacco product users (*p* = 0.0197). In contrast, no statistically significant difference in dependence severity was observed between heated tobacco product users and combustible cigarette users (*p* = 0.6775).

Multivariable linear regression, adjusting for potential confounders, identified distinct predictors of tobacco smoking. Parental smoking (aOR = 1.98; *p* < 0.001) and male gender (aOR = 2.13; *p* < 0.001) were associated with an increased likelihood of smoking. Conversely, primary education (aOR = 0.44; *p* < 0.001) and financial stability (aOR = 0.40; *p* = 0.010) were associated with a lower likelihood of tobacco use. Other variables, including place of residence, were not statistically significant in the adjusted model.

## 7. Discussion

The latest research from 2024 found that disposable e-cigarettes are the most used type of nicotine products by adolescents and young adults, while traditional cigarettes are used much less frequently. Foreign analyses, as well as our own research, showed that about one third of young people declaring the use of nicotine-containing products chose disposable e-cigarettes as the main form of nicotine intake. Traditional cigarettes were the least popular choice. These findings indicate a shift in nicotine consumption patterns among young people—instead of giving up nicotine, young people may be substituting combustible tobacco products with alternative nicotine delivery systems, which may contribute to the perception that these products are less harmful than conventional cigarettes. This pattern may contribute to sustained nicotine exposure and the development of nicotine addiction and its negative health effects. The cited research from the USA draws attention to the reduction in the number of regular smokers among young people, thanks to effective educational campaigns conducted on a massive scale. At the same time, despite the decline in the overall number of e-cigarette users in recent years, disposable products still dominate the preferences of young people, which poses a challenge to public health prevention efforts [14,15].

Understanding the factors associated with nicotine use is essential for developing effective prevention strategies. Our study findings indicated that factors such as urban residence, lower financial stability, and having a smoking parent during childhood were associated with higher prevalence of nicotine product use.

Socio-economic position influenced the vaping/smoking behaviour of the surveyed youth. A 2024 study by the University of Waterloo found that teens with lower family income were more likely to use nicotine products. This was influenced by, m.in factors, related to the possession of pocket money, allowing the purchase of the aforementioned products, despite the lower financial status [16]. A study published by the American Heart Association found that people from families with lower social status and parents with lower education were more likely to smoke/vape, and teenagers from higher-income families were more likely to perceive nicotine products as harmful to health [17]. In studies conducted in the UK and the US, parents smoking, especially when they both smoked or vaped, significantly increased the likelihood that their children would also use nicotine-containing products. The normalization of nicotine use at home strongly influences adolescents’ decisions to start vaping [18,19].

The type of nicotine product used was also compared with financial stability. Studies in the US showed that people with lower incomes were more likely to smoke traditional cigarettes, while having higher incomes was associated with more frequent use of electronic cigarettes [20]. Our findings suggest that participants with less stable financial backgrounds reported higher rates of traditional cigarette use. This could potentially be related to their lower cost and higher accessibility, although our study did not specifically investigate these factors. Conversely, those with more stable financial backgrounds showed a preference for disposable e-cigarettes; this pattern might be influenced by the perception of these products as more fashionable or less harmful, though further research is needed to confirm these underlying motivations.

The presented results highlight the importance of taking into account both financial and family factors in actions aimed at reducing the use of nicotine products among young people. Prevention strategies may require educating, both parents and children, about the risks associated with the use of nicotine-containing devices, especially in lower-income families where stress and lack of health education pose additional challenges.

There are gender differences in the use of e-cigarettes among young people, which is revealed both by our research in southern Polish and by data from other countries. Men tend to start using nicotine-containing products earlier and are more likely to become regular users compared to women [21,22]. It is also worrying that in an analysis carried out by the WHO, among young people in 17 European research centres showed that in the studied population of men, the highest percentage of electronic cigarette users was in Poland, similarly in the studied population of women, which definitely distinguishes Poland from other countries [21].

The results presented above point to the need for a more differentiated approach to education and prevention of e-cigarette use among adolescents and adolescents. Gender differences in the initiation and regularity of use of these products suggest that health campaigns should be tailored to specific groups, taking into account the different motivations and perceptions of risk between boys and girls.

American research, unlike ours, showed that young people from rural areas were more likely to use e-cigarettes compared to their urban peers [23,24,25]. While our study observed higher usage in urban settings, we did not collect specific data on retail density or local health policy efficacy. Therefore, the observed differences might be hypothesized to result from disparities in the accessibility of nicotine products between Polish rural and urban areas. Another potential explanation, which warrants further investigation, is that U.S. urban environments may benefit from more effective public health campaigns or stricter enforcement of sales regulations compared to Polish settings. These interpretations remain speculative and should be addressed in future studies designed to examine the environmental determinants of nicotine use.

These complex interactions between social, economic and demographic factors require further analysis in the context of effective prevention and education strategies.

Respondents used nicotine products for the first time at the age of 15.76 years on average. An analysis conducted in the USA in the years 2013–2017 in the PATH study shows that the most dynamic increase in the use of e-cigarettes was observed between the ages of 18 and 19 [26]. The data presented above indicate that e-cigarettes constitute a common source of initial nicotine exposure among young people entering adulthood. Additionally, the results of our study suggest that the Polish legislation prohibiting the sale of nicotine-containing products to minors has not been fully effective in preventing access to these products.

It is also worrying that the so-called “dual use”, i.e., the simultaneous use of different nicotine products, is becoming more and more popular. Many young people who mainly use e-cigarettes also use other tobacco products, including cigarettes and heated tobacco products [27]. E-cigarettes, due to their pleasant smell and convenience of use, are more commonly used at home, while traditional cigarettes, less practical to use, are mainly smoked outdoors. This creates comfort for youngsters because parents are unable to smell after a vaping session [28].

In our study, smokers, both traditional cigarettes and e-cigarettes, accounted for as much as 14% of the surveyed group, while almost 1% of respondents used most of the available nicotine products. These are very worrying data due to the fact that “dual use” is a relatively new phenomenon associated with a difficult to predict health threat. Currently, we are well aware of the negative health effects of traditional cigarettes. However, the combination of traditional smoking with the use of e-cigarettes, which can be used almost all the time at home, outdoors, at work or while driving, significantly increases exposure to harmful substances contained in these products. Recently, researchers published a report in the Journal of Oncology Research and Therapy that the risk of developing lung cancer was four times higher in people who combined vaping and smoking, compared to those who only smoked [29]. These data are alarming and clearly indicate the need to reduce the phenomenon of “dual use” as much as possible.

Reports about the addictive potential of e-cigarettes are also alarming. According to current data, these devices are more addictive than traditional cigarettes [30]. This is due, among other things, to the ease of use, attractive flavors, high nicotine concentration, and the ability to vape frequently even indoors. It is a matter of concern that e-cigarettes are often seen as a less harmful alternative, which encourages experimentation by people who would never have used for traditional cigarettes, leading to an escalation of the problem of nicotine addiction among young people, whose addiction levels are constantly increasing [31].

In our study, young men demonstrated significantly higher average nicotine dependence scores than young women. However, there is a lack of current studies that compare the level of nicotine addiction using the Fagerstrom Test in adolescents between boys and girls. In the adult population, men showed higher levels of addiction, but the data do not directly refer to adolescents [32]. Further research is needed in this area to understand the mechanisms of addiction among young adults and to introduce effective prevention solutions.

Few studies also examine the relationship between the stage of education and the degree of nicotine addiction. In our study, the lowest levels of addiction were shown by people attending university. A study conducted in 12 low-income countries showed that longer education was associated with lower nicotine consumption [33]. A Finnish study, on the other hand, showed that respondents with higher education were more likely to quit smoking compared to those with primary education [34]. This indicates the importance of both conducting educational activities and providing support in the fight against addiction of people with lower education.

Based on the presented results and analyses, it can be concluded that the problem of nicotine use by adolescents and young adults is a multidimensional and increasingly complex phenomenon. With the growing popularity of disposable e-cigarettes, which are perceived as less harmful and more readily available, it becomes crucial to understand that this is not just the replacement of one addiction with another, but often the beginning of an intense addiction.

The complexity of the problem is also underlined by socio-economic and family factors, such as financial status, place of residence, education and smoking parents, which have a significant impact on the risk of nicotine use. In addition, the growing phenomenon of “dual use” and alarming data on the higher risk of diseases such as lung cancer require immediate and effective action.

Therefore, effective prevention and education strategies must be comprehensive, tailored to specific groups (including gender differences) and targeted not only at young people but also at their parents. Only in this way will it be possible to reduce the phenomenon of nicotine addiction in this age group, which is crucial for public health.

## 8. Study Limitations

Several limitations of this study should be acknowledged. Firstly, due to the cross-sectional survey design, our findings are limited to identifying statistical associations between variables, and no direct causal relationships can be established.

Secondly, the study relied on a convenience sampling method within a single region in southern Poland, which limits the representativeness of the group. While this approach provides valuable regional insights into contemporary nicotine consumption patterns among adolescents and young adults, the geographical focus and non-probabilistic sampling should be taken into account when interpreting the generalizability of the results.

Thirdly, the survey instrument did not undergo a formal, large-scale statistical pilot study prior to distribution, which limited the preliminary psychometric evaluation of the custom questionnaire items. Regarding the assessment of electronic cigarette dependence, our survey utilized a framework closely modeled after the original, established e-FTND. While this adapted version was not independently validated, its structure remains highly consistent with the original psychometric tool.

Lastly, our methodology compares dependence scores exclusively among individuals who are current, active users of these specific products. Consequently, these data reflect the severity of dependence within each group of consumers but cannot be used to establish or compare the absolute, inherent addictiveness of one product over another.

## 9. Conclusions

1. In the study group of adolescents and young adults, disposable e-cigarettes were the most used nicotine-containing product.

2. Parental smoking and male gender were positively associated with the likelihood of smoking, whereas primary education and financial stability were inversely associated with tobacco use.

3. Electronic cigarette users exhibited the highest average nicotine dependence scores. Multivariate analysis confirmed that male gender, the type of nicotine product used, and place of residence were independently associated with higher Fagerström test scores.

## Figures and Tables

**Table 1:** General characteristics of the study group.

Study Group *n* = 625			
General Data		*n*	%
Gender	Woman	350	56.00%
	Man	275	44.00%
Age	14–19	329	52.64%
	20–24	221	35.36%
	25–29	75	12.00%
Place of residence	Village	246	39.36%
	City	379	60.64%
Education	Professional	29	4.64%
	Basic	262	41.92%
	Medium	279	44.64%
	Higher non-medical	27	4.32%
	Higher Medical	28	4.48%
Financial stability	Less stable	34	5.44%
	Stable	365	58.40%
	More stable	226	36.16%
Contacts with nicotine	I smoke	212	33.92%
	I used to smoke	81	12.96%
	I once tried to smoke	147	23.52%
	Never have I ever smoked	185	29.60%
The current stage of education	Primary school	3	0.48%
	Vocational school	17	2.72%
	High School	237	37.92%
	Technical School	77	12.32%
	University	239	38.24%
	Work	52	8.32%
The main source of regularly taken nicotine	Disposable E-Cigarette	69	11.04%
	Standard e-cigarette	58	9.28%
	Tobacco Heater	50	8.00%
	Cigarettes	35	5.60%
	I don’t smoke, or I smoke occasionally	413	66.08%

Explanation of abbreviations: *n*—number.

**Table 2:** Characteristics of the study group taking into account occasional sources of nicotine intake (multiple selection).

Study Group *n* = 625		
Nicotine-Containing Products Used by Respondents Occasionally	*n*	%
Disposable E-Cigarette	156	24.96%
Cigarettes	107	17.12%
Tobacco Heater	72	11.52%
Standard e-cigarette	60	9.60%
Cigarillos	32	5.12%
Water pipes	28	4.48%
All of the above	5	0.80%
People smoking both e-cigarettes and traditional cigarettes	88	14.08%

Explanation of abbreviations: *n*—number.

**Table 3:** The association between sociodemographic and environmental factors and the regular use of nicotine-containing products by the test subjects.

Variable	Smoking *n* = 212		Non-Smoking *n* = 413		*p*	OR	95% CI
	*n*	%	*n*	%			
Cigarette smoking by parents during childhood							
Smoking parents	101	43.16%	133	56.84%	0.00016	1.92	1.37–2.68
Non-smoking parents	111	28.39%	280	71.61%		1	-
Financial stability							
Less stable	18	52.94%	16	47.06%	0.01178	1	-
Stable	110	30.14%	255	69.86%		1.15	0.51–2.58
More Stable	84	37.17%	142	62.83%		0.83	0.4–1.72
Place of residence							
Village	70	28.46%	176	71.54%	0.02008	0.66	0.46–0.94
City	142	37.47%	237	62.53%		1	-
Gender							
Male	115	41.82%	160	58.18%	0.00022	1.87	1.32–2.65
Female	97	27.71%	253	72.29%		1	-
Education							
Occupational	14	48.28%	15	51.72%	0.00461	1	-
Primary	70	26.72%	192	73.28%		0.39	0.17–0.91
Secondary education	101	36.20%	178	63.80%		0.61	0.27–1.38
Higher non-medical	13	48.15%	14	51.85%		0.99	0.35–2.82
Higher medical	14	50.00%	14	50.00%		1.07	0.38–3.02

Explanation of abbreviations: ***n***—number, ***p***—statistical significance, **OR**—Odds ratio, **CI**—Confidence Interval.

**Table 4:** Factors associated with current smoking status: Results from multivariable logistic regression analysis.

Variable	Smoking *n* = 212		Non-Smoking *n* = 413		Adjusted OR	95% CI	*p* Adjusted
	*n*	%	*n*	%			
Cigarette smoking by parents during childhood							
Smoking parents	101	43.16%	133	56.84%	1.979	1.38–2.84	0.0002
Non-smoking parents	111	28.39%	280	71.61%	1	-	-
Financial stability							
Less stable	18	52.94%	16	47.06%	1	-	-
Stable	110	30.14%	255	69.86%	0.3965	0.19–0.84	0.0098
More Stable	84	37.17%	142	62.83%	0.5173	0.24–1.12	0.4174
Place of residence							
Village	70	28.46%	176	71.54%	0.7011	0.49–1.01	0.0580
City	142	37.47%	237	62.53%	1	-	-
Gender							
Male	115	41.82%	160	58.18%	2.127	1.48–3.06	0.0000
Female	97	27.71%	253	72.29%	1	-	-
Education							
Occupational	14	48.28%	15	51.72%	1.578	0.52–4.79	0.0644
Primary	70	26.72%	192	73.28%	0.4353	0.29–1.00	0.0004
Secondary education	101	36.20%	178	63.80%	0.7361	0.32–1.67	0.4704
Higher non-medical	13	48.15%	14	51.85%	0.8016	0.27–2.42	0.9066
Higher medical	14	50.00%	14	50.00%	1	-	-

Explanation of abbreviations: ***n***—number, ***p***—statistical significance, **OR**—Odds ratio, **CI**—Confidence Interval.

**Table 5:** Differences in the age of nicotine initiation according to sociodemographic and environmental factors.

Study Group *n* = 625					
Variable		Nicotine Initiation Age			
		Median and 25th–75th Percentile	Minimum Value	U Mann–Whitney Test	Kruskal–Wallis Test
Total study group	-	16 (14–17)	7	-	-
Gender	Men	15 (14–17)	7	0.0035	-
	Women	16 (15–17)	8		
Regular smokers	Disposable E-Cigarette	16 (14–17)	9	-	0.2985
	Standard e-cigarette	16 (14–17)	12		
	Tobacco heater (e.g., IQOS)	16 (15–17)	10		
	Cigarettes	15 (14–16.5)	11		
Smoking parents	Parents who smoke	16 (14–17)	7	0.5725	-
	Non-smoking parents	16 (14.25–17)	8		

Explanation of abbreviations: ***n***—number.

**Table 6:** The distribution of the degree of nicotine dependence according to the type of nicotine-containing product used by the respondents.

Study Group *n* = 625							
Regular Users of Nicotine-Containing Products *n* = 213							
Variable	Nicotine-Containing Device						
Fagerström Test Result	E-Cigarettes		Heated Tobacco Products		Cigarettes		*p*
	*n*	%	*n*	%	*n*	%	
0 to 3	43	33.86%	23	46.00%	23	63.89%	0.00026
from 4 to 6	34	26.77%	20	40.00%	9	25.00%	
from 8 to 10	50	39.37%	7	14.00%	4	11.11%	

Explanation of abbreviations: ***n***—number, ***p***—statistical significance, Adjusted significance level: *p* < 0.0167.

**Table 7:** Association of individual variables with the Fagerström test results among the subjects.

Study Group *n* = 625			
Regular Users of Nicotine-Containing Products *n* = 213			
Variable	Fagerstrom Test Score (Average)	Kruskal–Wallis Test	U Mann–Whitney Test
Nicotine product used			
E-cigarettes	5.01	0.0001	-
Heated tobacco products	3.66		
Cigarettes	2.88		
Place of residence			
City	4.54	-	0.17
Village	3.9		
The current stage of education			
Primary school	9	0.0107	-
Vocational school	4.66		
High School	5.24		
Technical School	4.66		
University	3.61		
Work	4.64		
Financial stability			
Less stable	4.61	0.0778	-
Stable	3.88		
More stable	4.86		
Gender			
Man	5.1	-	<0.0001
Woman	3.41		

Explanation of abbreviations: ***n***—number, Adjusted significance level: *p* < 0.0167.

**Table 8:** Multivariate linear regression analysis of factors associated with Fagerstrom test scores.

Study Group *n* = 625				
Regular Users of Nicotine-Containing Products *n* = 213				
Independent Variable	Standardized Coefficient (β)	Standard Error β	*p*	95% CI
Gender	0.325	0.062	<0.001	0.2–0.45
Type of product	0.337	0.064	<0.001	0.21–0.46
Education	0.156	0.108	0.149	−0.06–0.37
Residence	0.145	0.063	0.023	0.02–0.27
Age	0.037	0.062	0.734	−0.08–0.16
Financial stability	0	0.064	0.997	−0.13–0.13

Explanation of abbreviations: ***n***—number, ***p***—statistical significance, **CI**—Confidence Interval. Model statistics: R^2^ = 0.230, Adjusted R^2^ = 0.207.

## Data Availability

The original contributions presented in this study are included in the article. Further inquiries can be directed to the corresponding author.
